# Enhancing Extensive and Remote LoRa Deployments through MEC-Powered Drone Gateways

**DOI:** 10.3390/s20154109

**Published:** 2020-07-23

**Authors:** Jorge Gallego-Madrid, Alejandro Molina-Zarca, Ramon Sanchez-Iborra, Jorge Bernal-Bernabe, José Santa, Pedro Miguel Ruiz, Antonio F. Skarmeta-Gómez

**Affiliations:** 1 Department of Information and Communication Engineering, University of Murcia, 30100 Murcia, Spain; jorgegm@um.es (J.G.-M.); alejandro.mzarca@um.es (A.M.-Z.); jorgebernal@um.es (J.B.-B.); pedrom@um.es (P.M.R.); skarmeta@um.es (A.F.S.-G.); 2 Department of Electronics, Computer Technology and Projects, Technical University of Cartagena, 30202 Cartagena, Spain; jose.santa@upct.es

**Keywords:** drones, LPWAN, NFV, MEC, LoRAWAN

## Abstract

The distribution of Internet of Things (IoT) devices in remote areas and the need for network resilience in such deployments is increasingly important in smart spaces covering scenarios, such as agriculture, forest, coast preservation, and connectivity survival against disasters. Although Low-Power Wide Area Network (LPWAN) technologies, like LoRa, support high connectivity ranges, communication paths can suffer from obstruction due to orography or buildings, and large areas are still difficult to cover with wired gateways, due to the lack of network or power infrastructure. The proposal presented herein proposes to mount LPWAN gateways in drones in order to generate airborne network segments providing enhanced connectivity to sensor nodes wherever needed. Our LoRa-drone gateways can be used either to collect data and then report them to the back-office directly, or store-carry-and-forward data until a proper communication link with the infrastructure network is available. The proposed architecture relies on Multi-Access Edge Computing (MEC) capabilities to host a virtualization platform on-board the drone, aiming at providing an intermediate processing layer that runs Virtualized Networking Functions (VNF). This way, both preprocessing or intelligent analytics can be locally performed, saving communications and memory resources. The contribution includes a system architecture that has been successfully validated through experimentation with a real test-bed and comprehensively evaluated through computer simulation. The results show significant communication improvements employing LoRa-drone gateways when compared to traditional fixed LoRa deployments in terms of link availability and covered areas, especially in vast monitored extensions, or at points with difficult access, such as rugged zones.

## 1. Introduction

The Internet of Things (IoT) is continuously evolving since its inception more than ten years ago. A new wave of technologies are revolutionizing this paradigm from different perspectives, hence enabling the development of novel services that are devoted to different fields of application, e.g., smart cities, smart agriculture, or Intelligent Transportation Systems (ITS), among many others [1,2].

Attending to communication technologies used in this field, Low-Power Wide Area Network (LPWAN) is a recently-arrived solution to the IoT ecosystem, which is receiving great attention these days [3]. Solutions that are based on this paradigm promise long transmission ranges (more than 15 km [4]) while preserving end-device battery. Both factors are really valued for deploying IoT networks in remote locations or with difficult access. LPWAN technologies may be classified in two different families: (i) solutions that are integrated within a cellular infrastructure, e.g., Narrow Band-Internet of Things (NB-IoT) and (ii) solutions that deploy their own infrastructure in an ad-hoc fashion, e.g., LoRa Wide Area Network (LoRaWAN). While NB-IoT is currently under deployment and it is an active development line within 3GPP, aligned to the progress towards 5G, technologies such as LoRaWAN are in a mature stage and its use has been generalized for boosting the development of IoT applications in different fields [5], given the added value of being licence-free in some cases.

At the computing plane involving IoT systems, novel approaches, such as fog computing or Multi-Access Edge Computing (MEC), are currently dealing with the challenge of providing edge computing capacities. Fog and MEC both propose to distribute the intelligence near the end device, instead of using a centralized approach, such as the strategy adopted in pure cloud computing. This paves the way for the development of a plethora of applications with stringent latency or context-awareness demands [5]. In addition, network softwarization and virtualization techniques further improve the management of IoT infrastructures when facing changing scenarios. Concretely, both Software Defined Networking (SDN) and Network Function Virtualization (NFV) concepts are being considered for evolving IoT networks to the next level. SDN allows for managing switching and routing nodes in a network remotely according to a set of configurable rules, and NFV enables the pure virtualization of these devices while using software units. It is, in fact, the dynamic nature of these technologies that is boosting the integration of IoT platforms within the ITS ecosystem, for instance. The first step in this line was to provide road vehicles with connectivity under the Internet of Vehicles (IoV), which is an IoT spin-off concept applied to the vehicle domain. Moreover, novel developments addressing the virtualization of the vehicle’s On-Board Units (OBUs) for its agile monitoring can be found in the literature [6]. But also the same OBUs can be used to host different Virtualized Networking Functions (VNFs), thanks to the processing capability of embedded computers, e.g., Raspberry Pi series.

Even though LPWANs can improve the connectivity of smart environments, it has been detected that there are still limitations when trying to cover large and remote areas without a pre-existing network infrastructure. This is the case of IoT deployments at sea, mountain, forest, or when facing disasters with cuts in telecommunications. A common scenario using IoT technologies is agricultural or environmental monitoring on remote areas, which can involve difficult access conditions and scarce connectivity possibilities. It is under these circumstances where the synergy between LPWAN and virtualization/MEC technologies, together with new ITS options, such as the use of drones, can better support the collection of sensor data by providing sporadic communication links.

Following the previous line, this work presents an integration of LPWAN and VNF capabilities in a mobile gateway to provide long-range connectivity to IoT devices placed in remote areas, avoiding the deployment a permanent network infrastructure. The mobile gateway is mounted on top a virtualization platform that is installed on-board an Unmanned Aerial Vehicle (UAV), i.e., a drone. It is provided with LoRaWAN communications and it is able to create a new network segment covering larger areas for collecting data from remote sensor devices. When considering the transmission ranges provided by LoRaWAN, namely, around 20 km with Line-of-Sight (LOS) [4]) and the high distances covered by common industrial drones, approximately 11 km in a 20 minutes flight at half their maximum speed, this solution may be valid for covering vast areas of dozens of hectares. Besides, the virtualization platform includes a series of dedicated virtualized processing functions, hence adopting a MEC architecture in order to perform computation tasks locally, i.e., without the support of the fixed infrastructure. This approach permits saving both communication and energy resources to the UAV’s OBU, which is able to command end-devices to adapt to changing conditions or carry out local data-processing operations. This proposal has been validated in a real deployment and its potential has been comprehensively tested via computer simulation.

The main contributions of this paper are the following:

- A mobile LoRaWAN gateway is devised and developed.
- A MEC-based and UAV-enabled network attachment architecture for sensory nodes is proposed and discussed.
- An on-board virtualization platform for hosting both networking and processing tasks is designed and implemented.
- The flying MEC virtualization platform hosting a LoRaWAN gateway is validated and evaluated in both experimental and simulation test-beds.

The rest of the document is organized, as follows. Section 2 explores the most relevant works in the addressed field. A system architecture is presented and discussed in Section 3. The implementation and trials are described in Section 4. Section 5 exposes and discusses the obtained results. Finally, Section 6 presents the conclusions and future research lines.

## 2. Related Work

UAVs are being extensively employed in deployments to cover remote areas with connectivity or as a complement of the fixed infrastructure in highly crowded situations [7]. A great number of recent works have been published in this field in the research literature [8,9]. In [10], the authors highlight the challenges in terms of connectivity in self-organized UAV deployments involving many UAVs. Attending to the contribution of the paper in the area of sensor interconnection using UAVs, in the following we focus on the application of these vehicles in IoT scenarios [11].

Providing enhanced network connectivity from the sky by means of aircrafts, altitude platforms, airships, and drones is getting more and more research attraction in wireless networks, as it provides a valuable mean to complement terrestrial communications. In this regard, authors of [12] designed and implemented a solution that deployed LTE base stations (RRHs) in aerial Helikites to serve users in the ground. Recently, Saraereh et al. [13] proposed a UAV-enabled LoRa network architecture and associated topology control algorithm for disaster management networks. In their solution, UAVs are used to establish a mesh network that acts a relying node between the base stations and IoT devices that are carried by firefighters. The proposed algorithm adapts the topology as the terrestrial LoRa nodes move on the field, keeping all of the elements of the UAV swarm in a compact formation, thereby improving the packet reception rate. Similarly, in [14], the authors proposed an algorithm to coordinate different drones with the aim of increasing average packet throughput and LOS connections. Nonetheless, our UAV-MEC for LoRaWANs are not intended to move constantly and update their moving directions following a control algorithm, but rather moving to a particular location reducing energy in transmissions and increasing packet delivery ratios. Likewise, in [15], Yuan et al. proposed an UAV swarming platform that uses LoRa, WiFi, and LTE to provide enhanced communications between the UAV swarm, while achieving ultra-reliable IoT communications. Nevertheless, their UAVs are not primarily intended to be used as MEC gateways to serve ground IoT devices.

There are some research works [16,17,18] that consider small UAVs (SUAVs) as programmable network platforms that are capable of executing virtual functions and services relying on NFV. However, they do not consider LPWAN technologies in the communications plane of the SUAV, as it is the focus of our work. Similarly, the authors in [19] propose a lightweight and modular SDN/NFV architecture for the migration of UAV-related network services; however, they do not address further communication issues with extra radio technologies in the UAV.

In [20], the authors propose a multi-UAV system and demonstrate its viability for different use cases, including disaster assistance, search and rescue and aerial monitoring, addressing the challenges of communications, coordination, and sensing. Nonetheless, they do not address the scalability issues and automatic deployment of virtual network functions in UAVs.

Particularly considering LPWAN technologies, the work in [21] uses LoRaWAN as a secondary telemetry communication channel for UAV delivery. The authors in [22] go a step further by using UAVs as LoRaWAN gateways, and propose energy efficiency functions for controlling the connectivity in the UAV, thereby optimizing battery resources for UAV-based surveillance. The set-up of a moving LoRaWAN gateway is also included in our work; however, as a key difference, we propose a MEC architecture powered by NFV/SDN technology to dynamically deploy VNF and multi-RAT technologies on the UAV. The same lack is found in [23], in which the authors propose a UAV prototype that mounts a LoRaWAN gateway to collect data from IoT-cabaple ground sensors in agriculture and forestry scenarios.

The first research works analyzing the benefits of using LoRa gateways on UAVs to increase signal coverage in urban and suburban environments have appeared recently [24]. In this particular contribution, the authors measure the performance of the communication channel with sensors by attending to RSS at the LoRa GW. However, evaluations do not consider challenging scenarios with orographies hindering communications, including mountains and elevated land masses, as we study in our work. In a recent work [25], the authors propose a network architecture for data gathering in agricultural applications, leveraging UAVs equipped with different radio technologies, such as LoRa and WiFi. However, in their experiments, they do not test the real influence of the UAV flying height, and do not assess the node-to-UAV LoRa communications performance.

The work presented in this paper presents a breakthrough in the area of sensor interconnection using IoT technologies, by using UAVs as flexible NFV-based nodes to move processing and communication capabilities near field deployments. This way, both LPWAN interconnection and MEC capabilities can be delegated to remote locations with connectivity restrictions due to lack of infrastructure and/or presenting geographical difficulties for long-range wireless connections. This synergy among NFV, MEC, UAV, and IoT allows us to outperform any previous work in the area, and the system is both simulated and validated under real settings with a reference deployment.

## 3. Architecture

**Figure 1:**
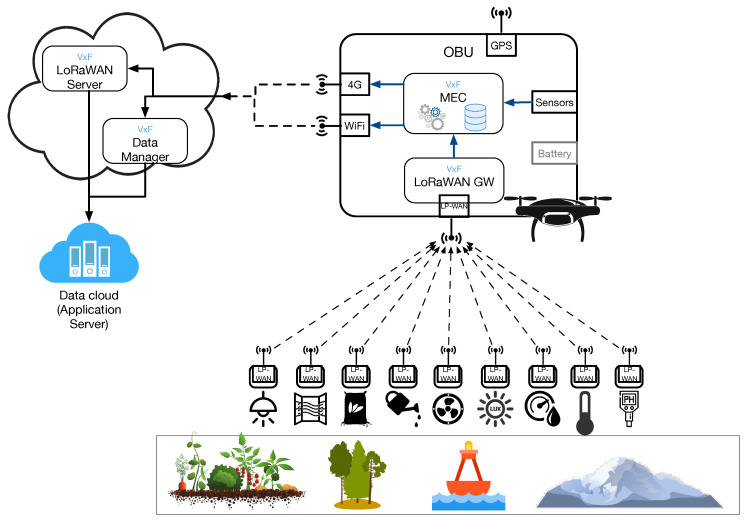
General architecture of the LoRa Wide Area Network (LoRaWAN) connectivity and Multi-Access Edge Computing (MEC) processing solution.

Figure 1 shows the overall scenario and solution of the proposal. As can be seen, the solution embraces a set of remote monitoring study cases. Under these scenarios, plants, soil, water, climate, weather, animals, and a vast set of “things” are supposed to be equipped with sensors attached to a low-energy communication node able to transmit data records using LPWAN communications. In particular, LoRaWAN is chosen as LPWAN technology, given its flexibility in the deployment, its wide adoption among the research community, and the availability of hardware modules.

Because, at these remote locations, there is no network deployment, or the installation of a LoRaWAN gateway would imply a significant cost without guaranteeing the interconnection of all the installed devices, it is proposed to use UAVs to collect data from time to time. The frequency of data harvesting depends on the concrete application, regarding the need for maintaining up-to-date information about the deployment. Upon the approximation of the UAV to the target area, data periodically sent by sensory nodes are collected. The LoRa-drone gateway can replace a compromised fixed LoRaWAN gateway, or be delivered to specific remote location to increase the network coverage of specific IoT devices.

The UAV is provided with an on-board unit (OBU) that offers the LoRa-drone gateway (GW) functionality and a set of software modules with extra MEC capabilities. These modules are given as Virtual Functions (VxF) deployed over a local Virtual Infrastructure Manager (VIM) or container platform, which gives a flexible solution to deploy OBU functionalities. Currently, the MEC VxF is in charge of pre-processing and filtering data records with the aim of saving local memory and forwarding data bundles to our local infrastructure, which finally reports data to a proper high-end data cloud with Big Data and analytics capabilities. Thus, the software of the LoRaWAN gateway (packet forwarder) is deployed as a VNF that can be instantiated on demand in the MEC node. Due to the constrained nature of the drone in terms of battery and maximum weight, the drone is equipped with a lightweight hardware solution, e.g., Raspberry board, capable of hosting VNFs implemented as lightweight virtualized services, such as lxc or docker containers.

The LoRa-drone gateway is a multi-RAT (Radio Access Technology) device that is endowed with at least two different wireless interfaces for backhaul and fronthaul connectivity. The fronthaul transceiver implements LoRa radio technology to communicate with the LoRa IoT devices, whereas the backhaul transceiver, e.g., 4G or WiFI, is intended to be used as channel to establish communication between the LoRaWAN gateway and the LoRaWAN Server (both downlink or uplink communications). Beyond those two interfaces, in our architecture, the MEC-powered LoRa-drone is equipped with a GPS module and UAV radio interface aimed to trace the route as well as manage drone telemetry.

When a wireless communication channel is available, the MEC VxF sends data records locally processed as they are available. However, under periods of no coverage with 4G or WiFi, the OBU performs a store-carry-and-forward function. Hence, the regular operation when the infrastructure is reachable follows the LoRaWAN specifications [26], and data are reported from the OBU to the LoRaWAN Server after preprocessing/filtering, acting as a common LoRaWAN gateway. On the contrary, when the store-carry-and-forward capability is needed, the OBU saves all data and reports them to the data cloud when infrastructure connectivity is available, bypassing the LoRaWAN Server. In this case, the proxy module that is in charge of this task is the Data Manager, as can be seen in Figure 1. The LoRaWAN Server and the Data Manager are also both deployed as VxFs under a VIM hosted in a server.

## 4. Implementation and Testbed

This section describes the implementation of the MEC-powered LoRa-drone gateway as well as the scenario and testbeds that have been designed to exhaustively validate the feasibility, benefits, and performance of the proposed architecture. The solution has been validated in both a real physical deployment and through computer simulations.

### 4.1. Lora-Drone Gateway Implementation

**Figure 2:**
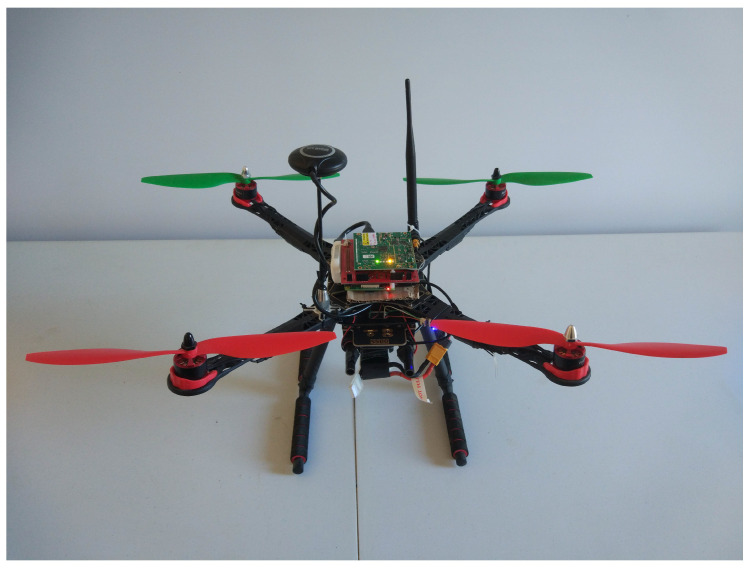
LoRa-drone gateway implementation used for testing.

Figure 2 shows the implemented LoRa-drone gateway. The main structure is composed by a plastic/carbon fiber 500 mm quad-copter frame and a 150 mm landing gear. The Power Distribution Board (PDB) is provided by the frame itself. The propulsion system consists of four 30 A Electronic Speed Controllers (ESCs) located under the arms, four 920 KV brush-less engines, and four propellers of 10in diameter and 4.5in pitch. The flight controller, located in the center of the frame, is a STM32-F722 (ST Microelectronics - Geneva, Switzerland) processor with dual gyros MPU6000 and ICM20602 (TDK - Tokyo, Japan). For the flight control software, it has been used the version 2.3.3 of INAVFlight.

We have also incorporated a GPS module with compass capabilities in order to improve the navigability of the LoRa-drone gateway. It is located at the top of a mast, covered by a plastic case. This component allows for us to enable features, like Return To Home (RTH), to make the LoRa-drone gateway land autonomously at the same place it took off. All these components are powered by an external four-cell battery of 14.8V and 2300mAh with a 45C discharge rate which provides between 10 and 15 minutes of flying time, depending on the payload, and that requires a charging time of around hour and a quarter. While in our experiments we maintain a pool of fully charged batteries that we use for repeating the experiments multiple times, commercial drone models usually board multiple batteries for extending flying time up to 38 min.

On-top the drone, it has been deployed the LoRaWAN gateway comprised of a LoRA concentrator, the omnidirectional antenna, as well as a lightweight single-board computer. Concretely, in our implementation, we have employed a Raspberry Pi III with an ic88a-spi LoRaWAN concentrator for the 868 Mhz band as well as an 1/2 wave 868Mhz omnidirectional antenna (5 dBi). The MEC node in the drone is implemented using Docker as virtualization environment to deploy the VNFs. The software deployed in the LoRaWAN gateway as VNF is The Things Network Zurich ic880a-gateway (https://github.com/ttn-zh/ic880a-gateway), which has been virtualised (docker) in order to allow an on-demand deployment of different services if necessary, therefore adopting a VNF-based approach. The backhaul connectivity with the LoRaWAN server is provided through a 4G adapter connected to the Raspberry Pi, although WiFi is also supported in current deployment. The gateway solution has been powered by a light weight battery of 10,400 mAh.

Table 1 shows the weight of each component, in order to calculate the Take-Off Weight (TOW) of the LoRa-drone gateway solution, which is defined as TOW=empty_UAV_weight+100_battery_weight+100_payload. In the formula, empty_UAV_weight represents the free of charge weight of the Drone (it includes GPS and GPS mast) (Drone in Table 1); 100_battery_weight represents the battery of the aircraft at 100% capacity (Drone battery in Table 1); and, 100_payload represents the whole payload of the aircraft (LoRaWAN GW, including the 4G modem + LoRaWAN GW battery in Table 1). In this case, the 500 mm drone frame with the engines is the heaviest part of the solution. As compared with the drone itself, the LoRaWAN gateway solution including the external battery represents 24.5% of the total weight, which remains within the limits of the theoretical Maximum Take-Off Weight (MTOW), since the maximum thrust capacity per engine provided by the engine manufacturer specification is around 890 g.

### 4.2. Evaluation Scenario

**Figure 3:**
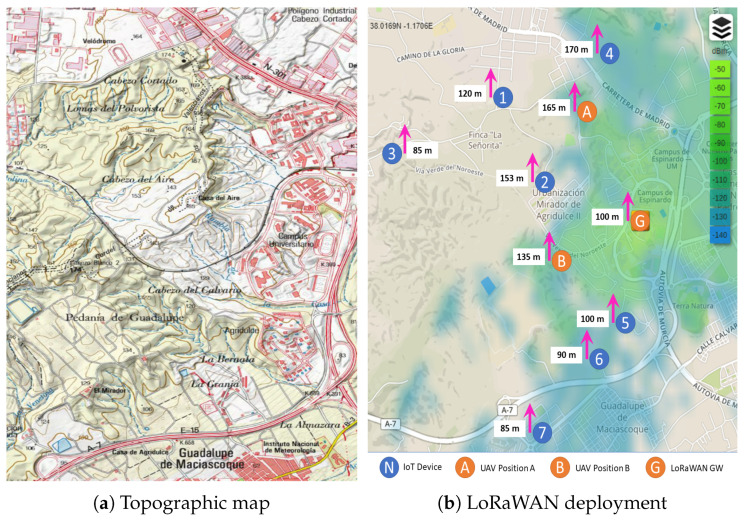
Maps of region under study, Espinardo Campus, University of Murcia (Spain).

As mentioned above, non-flat regions are difficult, or at least expensive, to be covered by a fixed communication infrastructure. It is in this type of scenarios where UAV-based communication systems may solve coverage troubles in a quick and efficient way. For that reason, for our validation and evaluation tests, we have chosen a real scenario with coverage issues due to terrain orography. This is the Espinardo Campus of the University of Murcia (Spain), which is placed on a hill with a peak elevation of ≈170 m above sea level on its northern area (Figure 3a) causing an important coverage obstruction to the communication systems placed at the southern area of the campus. Concretely, communication issues have been found for providing connectivity to the sensing elements installed in certain areas of interest when employing a preexistent LoRaWAN infrastructure, as discussed in the next paragraphs.

Prior to the experimental work, we have conducted a theoretical LoRaWAN coverage study in order to study the communications problems of the scenario. This has permitted us to finely identify coverage shadows due to the terrain orography in the specific areas of interest. The coverage study been conducted by mimicking the real LoRaWAN equipment described in the following section. We have made use of the cloudrf radio planning tool (http://cloudrf.com) and, as propagation model, the well-known Okumura–Hata one has been adopted. Subsequently, digital maps of the area are used, including orography information, to run the planning tool considering that the LoRaWAN gateway is placed in a telecommunications center deployed inside the campus, in the same location as it is currently installed and giving service. The obtained coverage map is shown in Figure 3b, where it can be seen how the LoRaWAN connectivity is lost in the northern highway and its surroundings, due to the propagation shadow provoked by the aforementioned terrain elevation. A similar lack of coverage is detected at the westside of the campus ring (marked as *Campus Universitario* in Figure 3b), caused again by another terrain elevation. The southern zone is hardly covered as well, due to the long distance to the gateway.

Figure 3b also shows some deployed IoT devices that are not properly covered by the current infrastructure. Specifically, we have identified seven different conflicting zones at different altitudes, in which the LoS is sporadically hampered by the terrain conditions. Each numbered circle represents an IoT device in a specific zone of interest to be monitored. The figures inside the white rectangles indicate the ground altitude above sea level for that point. Regarding the LoRaWAN base stations, the ‘G’ labeled circle indicates the current static LoRaWAN base station, while the ‘A’ and ‘B’ labeled circles indicate two different positions in which we deployed our mobile LoRaWAN UAV-based gateway in order to provide the conflicting IoT zones with connectivity. It is important to highlight not only the terrain obstacles between the base station and IoT devices, but also the altitude difference as well as the distance. For instance, the furthest point (3) is located around three kilometers away from the base station, inside a tunnel, with an altitude decrease of 15 m, whereas the nearest point (5) is located around one kilometer at the same level of altitude, but, in this case, behind a considerable group of buildings.

### 4.3. Testbeds Description

Two different testbeds have been considered for both validating and evaluating our proposal. Regarding validation, two real LoRaWAN deployments have been carried out for their comparison in the scenario presented above (University campus). First, the coverage provided by a fixed LoRaWAN infrastructure has been investigated; thereafter, a similar study has been done analyzing our implementation of the virtualization platform with both the LoRaWAN gateway and the MEC functions on-board a UAV. For the exhaustive evaluation, the second testbed involves a well-known network simulator when considering several configurations and conditions. In the following, we provide a detailed description of both testbeds.

#### 4.3.1. Validation Testbed in Real Physical Deployment

**Figure 4:**
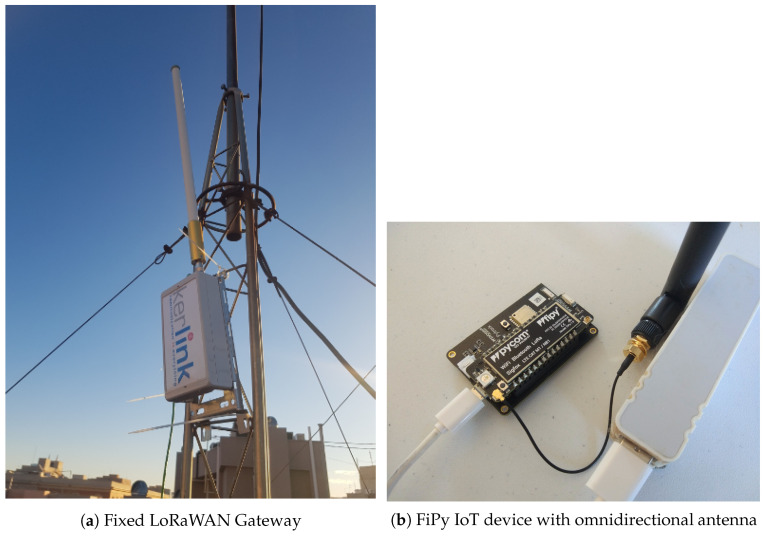
IoT and fixed LoRaWAN gateway used in testbeds.

First, we have tested the performance of a well-known LoRaWAN gateway installed approximately in the center of the Campus Ring (see Figure 3b). This fixed LoRaWAN setting allows comparing the benefits of using our LoRa-drone gateway against the traditional deployment. Concretely, we have employed the Kerlink Wirnet Station IoT outdoor LoRaWAN Gateway (https://www.kerlink.com/product/wirnet-station/) combined with an omnidirectional antenna with a gain of 5 dBi (Figure 4a). The transmission power was fixed to 0.025 W (14 dBm) in the 868 MHz frequency band. In turn, the end-device was a Pycom FiPy (https://pycom.io/product/fipy/) with the Semtech SX1272 long-range and low-power RF LoRa transceiver. An omnidirectional antenna with a gain of 2 dBi was attached to this device, which was placed on-board a car to make easier the testing campaign. Figure 4b shows the FiPy IoT device connected to a PyTrack expansion board that provides accurate GPS positioning. Both, FiPy and PyTrack are powered by an external battery of 1000 mAh.

As explained above, we have explored this scenario with the aim of evaluating the performance of the fixed LoRaWAN infrastructure that provides connectivity to the university campus in the northern region, when considering the theoretical coverage study to select and study problematic areas. For this purpose, we have deployed the ChirpStack LoRaWAN Network Server stack version 3 and we have developed a ping-pong application, which is executed in a virtual machine (server side) and in the IoT device. The IoT device ping application has been developed in Pycom Micropython 1.18.2.r7. When the end-device is powered, it joins to the LoRAWAN server and starts sending ping messages with a period of five seconds, alternating two different LoRaWAN data-rate (DR) configurations, namely DR0 and DR5, each five messages. Because DR0 max payload is 59 bytes, the maximum message size has been fixed to 50 bytes for all the tests. The messages are then composed by the tuple *(session_id, message_id, padding)*, where *session_id* identifies the current trial, *message_id* identifies the ping message in order to match it with the same *message_id* in the pong response, and the padding is auto-generated in order to complete the message size up to the maximum established. Each time a message is sent or received, the IoT device stores the tuple *(timestamp, session_id, type, message_id, rssi, dr, lat, long)* in a log file. Fields *session_id* and *message_id* have the same meaning than in the previous tuple. The *type* field identifies whether the log was generated by a *ping* or a *pong* message. The *rssi* field holds the received signal strength in dBm. Finally, the *lat* and *long* fields are the geographical coordinates of the end-node position. Regarding the server side application, it was developed in Python 3.7. When it is launched, a service is subscribed in the LoRaServer to the uplink events generated by registered IoT devices, hence it receives the uplink messages. When a ping message is received, it generates a pong message with the same *session_id* and *message_id*. It also generates the required padding in order to send a 50-byte message. In this case, data records contained in the sent and received messages are also stored in the log in order to verify the results of the session with the ones stored in the IoT device.

#### 4.3.2. Evaluation Testbed Through Simulation

In addition to the evaluation in a physical real deployment explained above, in order to conduct an exhaustive performance evaluation of the proposed architecture when considering different network conditions, we have employed the OMNeT++ network simulator (https://omnetpp.org/). This event-based simulator is powered by a series of libraries that permit simulating a great number of networking protocols and transmission technologies. Concretely, we have employed the FLoRa library (https://flora.aalto.fi/) supported by the well-known INET framework (https://inet.omnetpp.org/). FLoRa allows for the configuration and simulation of LoRa networks with end-devices, gateways, and a network server. It also supports Adaptive Data Rate (ADR) and collection of energy consumption statistics in the nodes.

We implemented a test-bed with one gateway and one end-device, both with the same specifications as the real ones described in the previous section, in order to perform the evaluation of the architecture in a realistic way. The position of the mote was fixed, while the gateway moved around the target area imitating the LoRa-drone gateway behaviour. The mote periodically transmits LoRa packets containing the maximum permissible payload for DR0, i.e., 51 bytes. To emulate the influence of real-world obstacles in communications, we used the log normal shadowing path loss model [27], as expressed in Equation (1). This model is an extension of the Friis free space model, and it is used to predict the propagation loss in complex environments, as it comprehends random shadowing effects due to signal blockage by terrain and buildings. In our case, the model is based on the real-world measurements taken from the outdoor experiments that were performed with the LoRa-drone gateway. Thus, we set 95 dB at a distance of 500 m as the base loss value $P_{L}\left(d_{0}\right)$. The path loss exponent *n* is what defines the environment fading characteristics in the simulation; hence, we used two values to characterize two different situations: 5 to create an urban scenario and 4 to simulate suburban conditions. Furthermore, to include random shadowing effects in the transmissions, we employed a Gaussian distributed random variable χ, centered in 8.5 dB with standard deviation 3.5 dB [27].

(1) $$\begin{array}{cc} \left[P_{L}\left(d\right)\right]dB=\left[P_{L}\left(d_{0}\right)\right]dB+10\;n\;\log_{10}\left(\frac{d}{d_{0}}\right)+\chi & for\;d_{f}\leq d_{0}\leq d \end{array}$$

For each scenario, six different configurations were considered, from Lora’s DR0 to DR5. Additionally, ten runs were carried out for each configuration, with the aim of analyzing the results with enough statistical confidence. Hence, we present the averaged values and the 95% confidence intervals (α = 0.05) in our results. As performance metrics, we considered the following: (i) the maximum coverage distance achieved as the UAV moves away from the mote, (ii) the energy consumption required to make a single transmission, and (iii) the Time on Air (ToA) of the transmitted packets.

## 5. Experiments and Results

### 5.1. Validation Results in Real Physical Deployment

We have performed a series of experiments in the same target areas identified in Figure 3 using both fixed and UAV-enabled architectures in order to validate our implemented architecture in the real deployment, as explained previously. First, we measured the Packet Delivery Ratio (PDR) for each conflicting IoT zone by using the static LoRaWAN deployment, i.e., Kerlink gateway, for two different data rates. These are, DR0 (250 bps) and DR5 (5 kbps). Subsequently, we repeated the experiments for the mobile architecture by flying the LoRa-drone gateway in two different points (see Figure 3b), increasing the elevation of the LoRa-drone gateway until the up-link PDR obtained with DR0 in each IoT zone is above 75% (Table 2). Thus, this table shows up-link and down-link PDRs obtained for both fixed and UAV cases when using DR0. In the case of the LoRa-drone gateway solution, it also provides the required altitude to keep up-link PDR percent above the mentioned threshold.

The results shows that the Kerlink base station was not able to receive messages from the IoT devices (0.0% up-link PDR), except for two locations: number 4, which is an elevated area, where 80% of the up-link messages were received properly, but all down-link messages were lost; and, number 5, which is the nearest point to the station and, at the same altitude, from where 40% of the up-link messages were received properly as well as 62.5% of the down-link messages. This poor performance of the fixed infrastructure calls for a solution that could increase the reception level, as explained above.

Regarding the LoRa-drone gateway solution, we obtained a significant improvement as compared to the previous case. For all of the locations except one, namely, point 3, the bi-directional communication was performed successfully. In fact, for all points, we achieved up-link PDR results above 75% and at five locations we achieved down-link PDR results above this figure. At location 3, it was required to elevate the LoRa-drone gateway up to 60 m, unlike the rest of cases, in which 10-35 m were enough to ensure the up-link communication threshold. This was due to a longer communication distance between location 3 and the drone position as well as additional orography difficulties.

When considering the tests for DR5, as expected, the results are significantly worse as compared to DR0, due to the increase of data rate and the consequent robustness loss. Table 3 shows the PDR values obtained for this set of experiments. For the Kerlink deployment, as a difference to the previous case, we only had one location with positive up-link PDR results, point 4, where only 15% of up-link messages were properly received. For the LoRa-drone gateway solution, we still had bi-directional communication in most of the points, but now the up-link PDR has decreased below 75% in all points except number 4 and 5. Even so, the improvement with respect to the fixed architecture is notable.

### 5.2. Performance Evaluation Results Through Simulation

**Figure 5:**
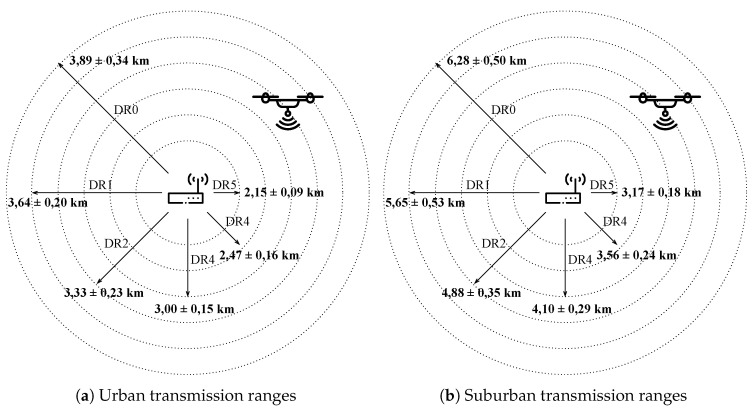
Transmission ranges in urban and suburban scenarios.

With the aim of evaluating the proposed system in an extensive way, we have also performed multiple experiments in the simulation environment. Firstly, we have evaluated the maximum transmission distance supported by the diverse LoRa’s DRs in two different scenarios, namely, urban and suburban settings, which have been characterized by means of the configurable propagation model described above. As shown in Figure 5, we have obtained shorter transmission ranges in the case of the urban scenario, due to the greater fading effect introduced by the propagation model. As compared to the transmission distances attained in the suburban scenario, they decrease by a factor of 1.5 approximately. Contrasting these outcomes with those experimentally obtained in a previous work [4], we prove the validity of our simulation framework. Besides, the impact of employing different LoRa configurations, i.e., DR, is also relevant. It is important to note that the use of high DRs, which is the desirable situation, as discussed later, is only possible at the closest end-device locations. This is a clear drawback of fixed LoRaWAN deployments that can be overcome by the use of a mobile flying gateway.

**Figure 6:**
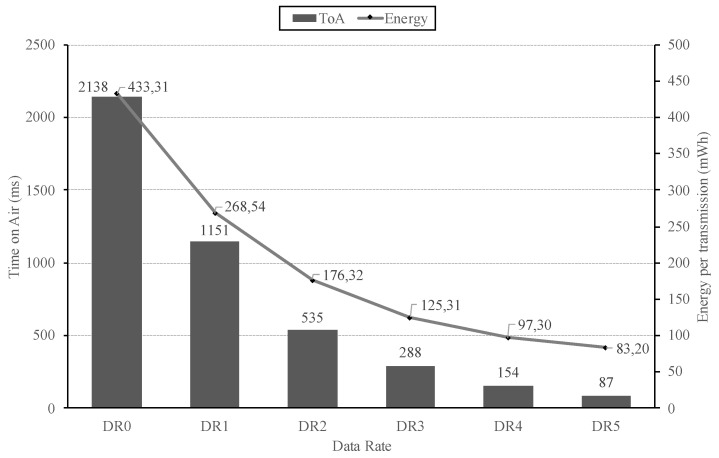
Time on Air and Energy per transmission for each data-rate (DR).

The advantages of using high DRs regarding energy consumption are shown in Figure 6. This figure presents the ToA and required energy per transmission obtained in the evaluated LoRa configurations. As aforementioned, the use of high DRs is preferred, as they permit reducing the transmission’s ToA and, consequently, the power consumption linked to the transmission. Both trends can be clearly seen by observing the figure and, as an example, employing DR5 instead of DR0 permits a decrease in power consumption by 500%. However, increasing the transmission DR leads to a decrease on the link robustness; hence, the distance between both extremes should be shortened, as discussed previously. In any case, this approach permits increasing the efficiency and data rate of communications as well as the end-device battery life.

From the above results, it is worth highlighting the communication improvements obtained by using a mobile LoRaWAN-dron gateway able to move closer to end-devices. Both experimental and simulation outcomes showcase the advantage of employing LoRa-drone gateways for vast monitored extensions, e.g., smart agriculture or farming, or at locations with difficult access, such as rugged areas.

## 6. Conclusions

This paper has presented the design, implementation, and evaluation of a novel architecture for enhancing LoRAWAN deployments by employing a LoRa-drone gateway. The solution allows the move the LoRaWAN gateway close to IoT end-devices to improve coverage wherever and whenever needed, such as emergency situation support or gaining connectivity at locations with difficult access, e.g., coverage obstructions due to orography or buildings.

The proposed LoRa-drone gateway implementation has been validated in a real physical LoRaWAN deployment, when comparing the benefits against fixed settings. The results show significant communications improvements of our solution as compared to traditional deployments, substantially increasing packet deliver ratios. In addition, extensive computer simulations have been conducted, showing performance benefits in terms of power consumption reduction and ToA when using the LoRa-drone gateway, thereby increasing the efficiency and data-rates of communications. The advantages are especially relevant in large monitored extensions, or at locations with difficult access, such as rugged areas.

As future work, we envisage extending the architecture through Software Defined Networking (SDN) and Artificial Intelligence-based approaches to dynamically and optimally reconfigure LPWAN networks and deploying LoRa-drone gateways on demand, according to the actual context or situation.

## Figures and Tables

**Table 1:** TOW of our LoRaWAN-drone gateway solution.

Drone (g)	Drone Battery (g)	LoRaWAN GW (g)	LoRaWAN Gateway (GW) Battery (g)	TOW (g)
893	231	177	189	1490

**Table 2:** PDR results for LoRa DR0.

	Fixed Gateway		LoRa-Drone Gateway		
Location	UL PDR (%)	DL PDR (%)	UL PDR (%)	DL PDR (%)	Altitude (m)
1	0.0	-	77.78	64.29	35
2	0.0	-	80.0	93.75	10
3	0.0	-	80.0	0.0	60
4	80.0	0.0	93.33	92.86	10
5	40.0	62.5	96.43	100	10
6	0.0	-	94.44	82.38	15
7	0.0	-	96.36	78.95	15

**Table 3:** PDR results for LoRa DR5.

	Fixed Gateway		LoRa-Drone Gateway		
Location	UL PDR (%)	DL PDR (%)	UL PDR (%)	DL PDR (%)	Altitude (m)
1	0.0	-	46.67	0.0	35
2	0.0	-	70.0	64.29	10
3	0.0	-	40.0	0.0	60
4	15	0.0	86.67	92.31	10
5	0.0	-	92.86	100	10
6	0.0	-	50.0	25.0	15
7	0.0	-	32.0	75.0	15
